# 3D-Printed Geopolymer Composite Truss Beam: Experimental Verification of Manufacturing and Load-Bearing Capacity in Four-Point Bending

**DOI:** 10.3390/ma19173632

**Published:** 2026-08-26

**Authors:** Vojtěch Jan Stoklasa, Vladislav Bureš, Oto Melter, David Čítek, Petr Zelený, Piotr Łoś, Katarzyna Ewa Łoś

**Affiliations:** 1 Faculty of Arts and Architecture, Technical University of Liberec, 461 17 Liberec, Czech Republic; vladislav.bures@tul.cz; 2 Klokner Institute, Czech Technical University in Prague, 166 08 Prague, Czech Republic; oto.melter@cvut.cz (O.M.); david.citek@cvut.cz (D.Č.); 3 Faculty of Mechanical Engineering, Technical University of Liberec, 461 17 Liberec, Czech Republic; petr.zeleny@tul.cz; 4 Polytechnic Faculty, University of Kalisz, Pl. Wojciecha Bogusławskiego 2, 62-800 Kalisz, Poland; piozyt@gmail.com

**Keywords:** 3D printing, geopolymer, truss beam, four-point bending, reinforcement, buildability, mechanical failure

## Abstract

Geopolymers represent a promising material platform for extrusion-based 3D printing; however, current research remains largely focused on mix design, rheology, printability, buildability, and the relationship between process parameters and the resulting microstructure. This article therefore compares the behaviour of two 3D-printed geopolymer composite truss beams with reference cementitious composite beams developed within the 3D STAR project. The geopolymer elements, 2932 mm long, were designed for the same material volume and target geometry as the reference CC element; however, because of mixture spreading, they reached cross-sections of only approximately 140/250 mm and 170/250 mm. The first beam was printed without setting acceleration, while the second was locally treated with a hot-air gun. In four-point bending, GC-2 was loaded first and reached 22 kN, while GC-1 was loaded second and reached 29 kN; the reference cementitious beams reached 31 and 40 kN. The CC elements failed by rupture of the tensile reinforcement, while the GC elements failed by joint failure followed by deformation and local disintegration of the composite. The study thus shows that the main difference between the two systems lies not only in the achieved load-bearing capacity, but also in stiffness, the shape of the load-displacement diagrams, and the failure mechanism.

## 1. Introduction

The abbreviations GC for geopolymer composite and CC for cementitious composite are used throughout the text. Additive manufacturing with CC and GC is an established direction in construction research. Current literature on GC mainly addresses mix design, rheology, extrudability, buildability, shape stability, interlayer bonding, and the relationship between process parameters and microstructure [1,2,3,4,5,6,7]. The assessment of real structural elements, the effect of layering on load-bearing capacity, and the integration of reinforcement into continuous printing are less unified [6,8,9,10,11,12].

For structural application, the decisive question is whether a load-bearing element can be reliably printed, reinforced, and verified from the material. In truss geometry, the joints, interaction with reinforcement, and preservation of the cross-section during printing are particularly important [8,10,11].

In previous research, a 3D-printed truss floor beam made of CC was developed. In four-point bending, it failed only after rupture of the tensile reinforcement, not by failure of layers or joints [10]. This created a reference framework for verifying the same structural concept using GC.

The GC mixture used in this study was lightweighted with 3M microspheres. These improve printing properties, reduce the sagging of lower layers, and decrease dead load; possible thermal-insulation and fire-resistance benefits were not evaluated in this work. The aim was not to prove the general superiority of GC, but to verify this particular mixture in a reinforced structural element.

The aim was to verify the printing and four-point bending behaviour of a GC beam whose shape was derived from a previously tested CC beam. The text focuses on structural behaviour; material data are provided only for the interpretation of results.

## 2. Materials and Methods

### 2.1. Research Concept

The research distinguishes between the material level (mixture composition, fresh behaviour, mechanical properties, and printing limits) and the structural level (manufacturing, reinforcement, and loading of a real beam).

This article focuses on the structural level. The material characteristics serve only to explain the geometry, printing strategy, and failure of the elements. This focus responds to the predominance of studies on mixtures and fresh behaviour compared with the smaller number of structural demonstrators [2,3,4,5,6,7].

### 2.2. GC Mixture Used

A GC mixture with which the team had the greatest previous experience was used. Its composition, preparation, and comparison of technological, density, and mechanical parameters of GC and CC are given in Table 1, Table 2 and Table 3.

The geometry of test specimens, mixing, and testing conditions are given in Table 1.

The lightweight filler was 3M K15 Glass Bubbles (3M Company, St. Paul, MN, USA; hollow soda-lime borosilicate microspheres; median diameter 60 µm, true density 0.15 g/cm^3^, isostatic crush strength 2.07 MPa) [13].

**Table 2:** Technological and density characteristics of GC and CC.

Parameter	GC	CC
Mixing time	approx. 45 min	–
Workability after mixing	approx. 60 min + approx. 30 min conditionally	–
Setting-acceleration method	without chemical accelerator; second beam locally hot air	reported 17% aqueous aluminium sulfate solution (Ekosal L; STACHEMA CZ s.r.o., Kolín-Zibohlavy, Czech Republic); added in the print head immediately before the nozzle; mixed by a rotating spindle inside the print head [14]
Dry bulk density [kg/m^3^]	1250	–
Wet bulk density [kg/m^3^]	1515	≈2400 (indicative value for dense CC)

#### Fresh-State Observations and Limitations

The operator-observed workability window was about 60 min, with a further ~30 min only conditionally usable as consistency changed. No rheometer measurements of yield stress, viscosity, thixotropy, structuration rate, early load-bearing capacity, or interlayer bond were performed; fresh-state behaviour is therefore reported qualitatively.

**Table 3:** Mechanical properties of GC and CC.

Property	1 Day		7 Days		28 Days		90 Days	
	GC	CC	GC	CC	GC	CC	GC	CC
Shrinkage [mm/m]	−9.55 ±0.76	–	−16.9 ± 0.62	−0.84	−16.8 ±0.60	−1.18	−16.4 ±0.61	−1.5
Compressive strength [MPa]	9.9 ±0.30	10	24.7 ±0.83	41.5	26.5 ±1.96	64.5	25.1 ±1.11	76.5
Flexural tensile strength [MPa]	2.7 ±0.31	3	7.4 ±1.03	8.3	9.4 ±0.49	11.1	9.1 ±0.64	12.2
Static modulus of elasticity [GPa]	1.1 ±0.04	–	3.1 ±0.33	–	3.7 ±0.56	32.1	3.8 ±0.18	–

Note: GC values are mean ± sample standard deviation (*n* = 3). Archived CC data were available only as the reported point values.

### 2.3. Reference CC Beam

**Figure 1:**
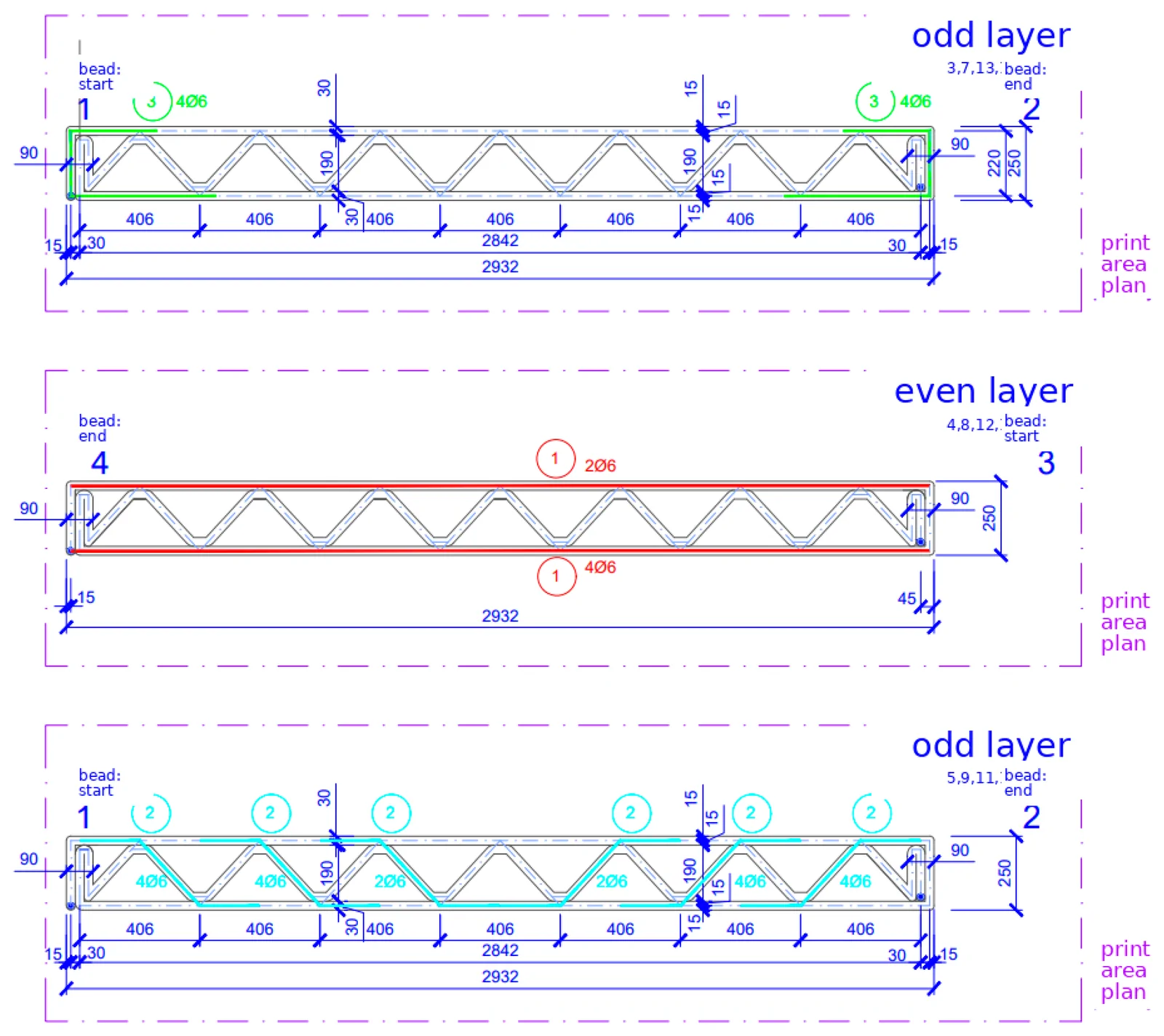
Reference CC truss beam: geometry and layer scheme. Dimensions in mm.

**Figure 2:**
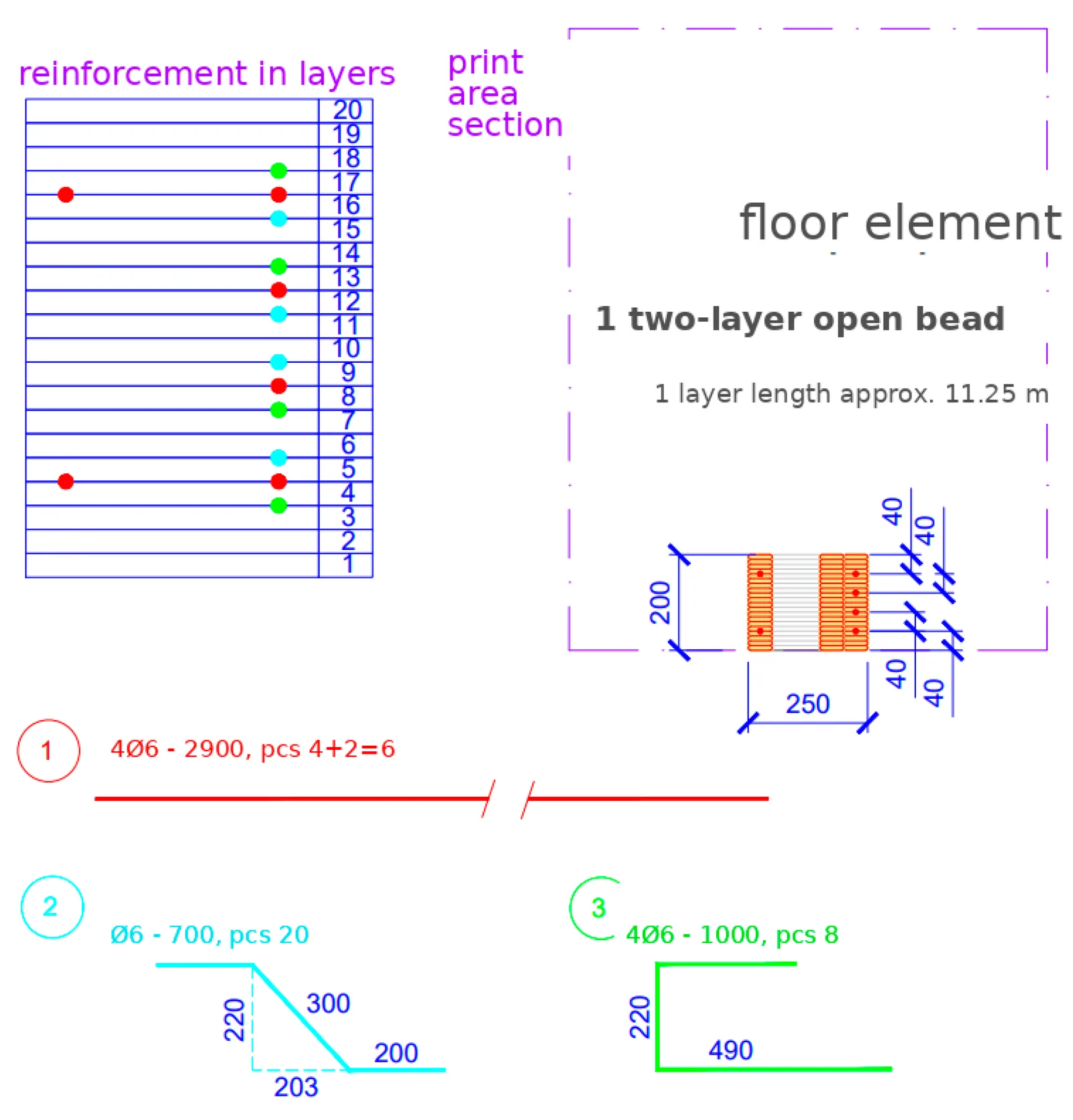
Reference CC truss beam: cross-section and reinforcement details.

The reference CC beam was taken from the 3D STAR project [14]. Its geometry and reinforcement are shown in Figure 1 and Figure 2, while material and printing parameters are summarized in Table 2, Table 3 and Table 4. During printing, liquid Ekosal L (STACHEMA CZ s.r.o., Kolín-Zibohlavy, Czech Republic; 17% aqueous aluminium sulfate) was dosed into the print head immediately before the nozzle. A rotating spindle mixed the accelerator with the CC before extrusion, so only the small volume in the head was accelerated. The load-displacement response is discussed in Section 3.

### 2.4. Geometry, Printing, and Testing of GC Beams

Two GC beams were printed with the same design volume, target geometry, and reinforcement arrangement as the CC reference. All beams used B500B bars (fyk = 500 MPa, Ø6 mm): four longitudinal bars in the bottom chord, two in the top chord, and four bars in the tension diagonals; compression diagonals were unreinforced. Reinforcement was inserted manually between printed layers.

The target print height was 200 mm and the target bead width 30 mm; achieved dimensions are summarized in Table 4.

Printing proceeded at approximately 1.3–2.9 m/min for GC-1 and 0.9–2.1 m/min for GC-2, with speed adjusted to mixture consistency. GC-1 was printed without acceleration. For GC-2, each layer was manually heated before the next layer using a Wagner FURNO 750 heat gun (J. Wagner GmbH, Markdorf, Germany) at maximum setting (630 °C; airflow 820 L/min). The effect was limited, especially compared with earlier trials using a 6 mm nozzle.

GC was manually charged into an open print head; a rotating spindle provided extrusion. No pump or closed hose feed was used because of the risk of the geopolymer setting in the delivery system.

The toolpaths comprised 40 nominal layers at 5 mm vertical increments. Programmed feed rates were 4.2 m/min for GC-1 and 3.0 m/min for GC-2; actual speed ranges are given in Table 4. The beam geometry used a 406 mm panel pitch, 250 mm overall structural depth and 220 mm vertical chord-centre distance.

**Figure 3:**
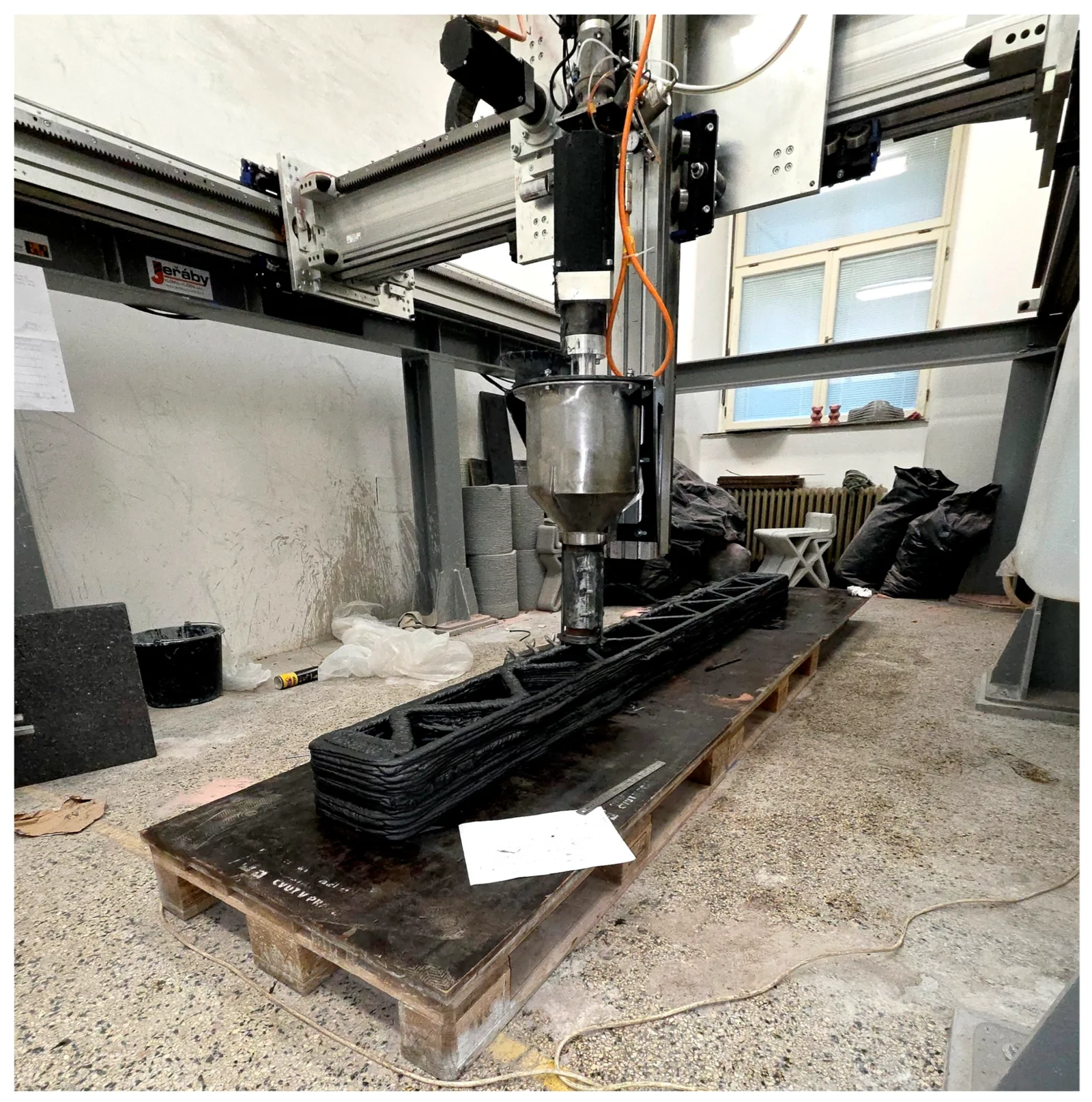
Printing of GC-1 without setting acceleration achieved width 140 mm.

**Figure 4:**
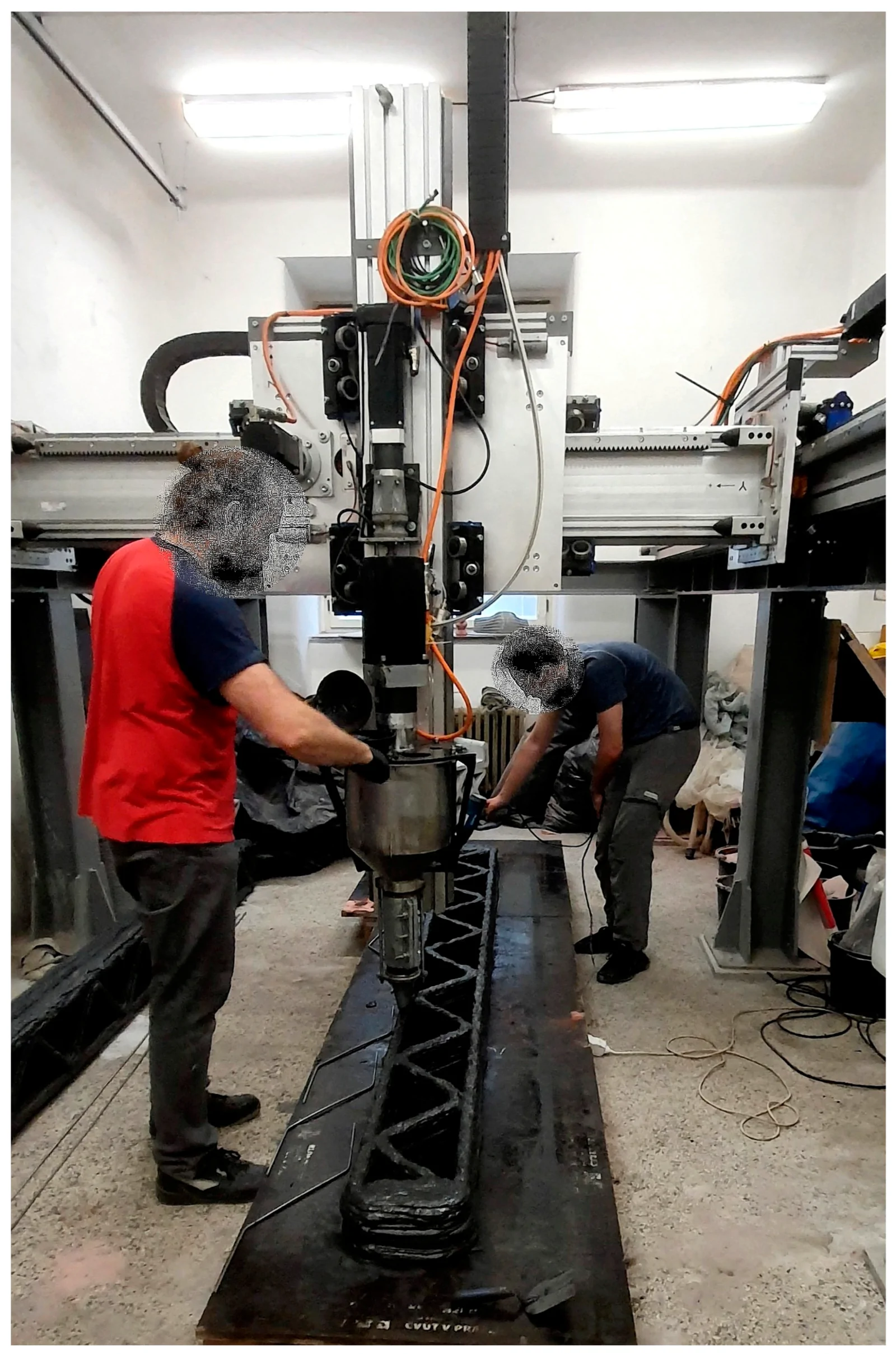
Printing of GC-2 with local interlayer hot-air treatment.

**Figure 5:**
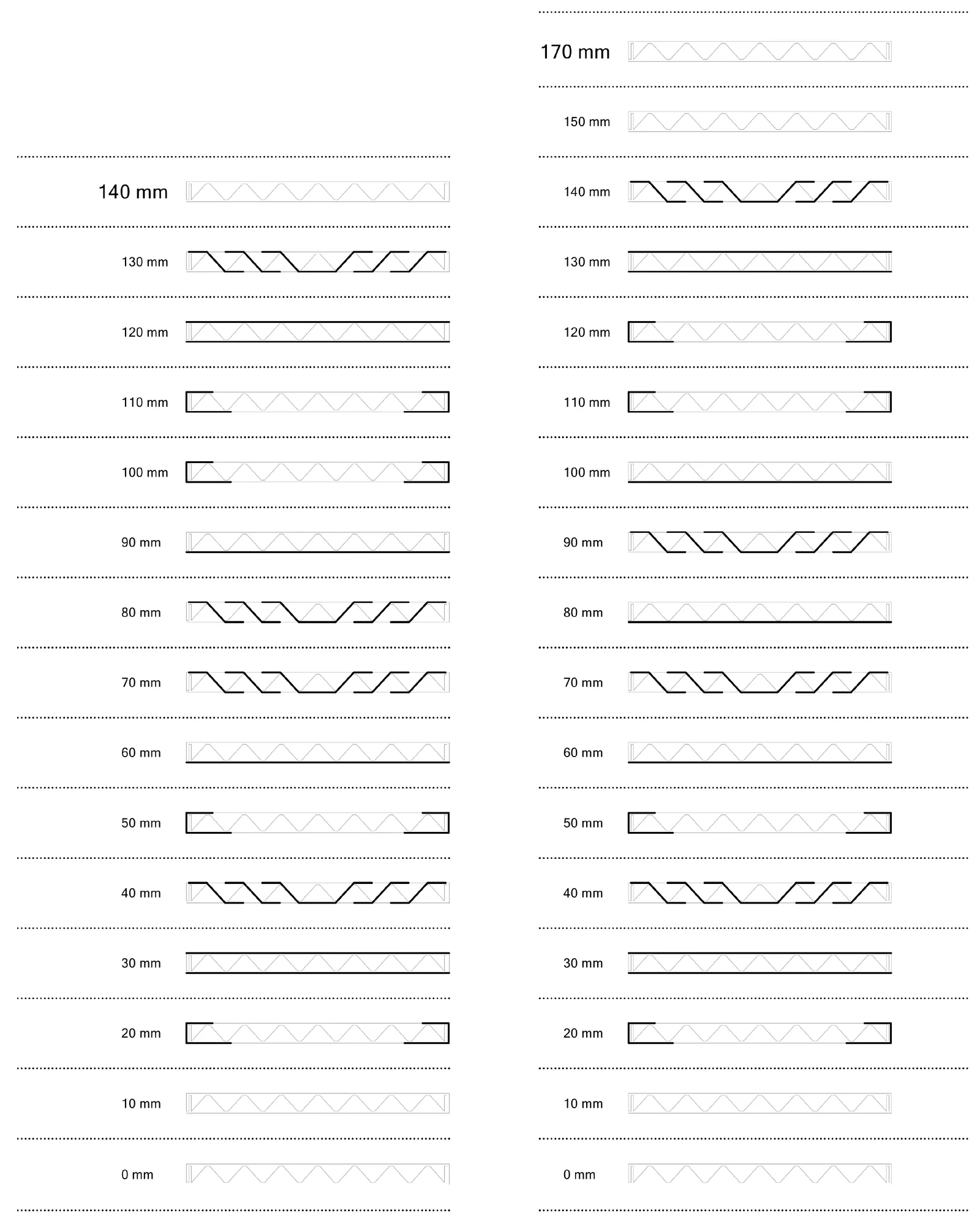
Actual reinforcement layouts in the beams: (**left**) GC-1 (achieved width 140 mm); (**right**) GC-2 (achieved width 170 mm). Dimensions in mm.

After printing, both beams were stored indoors at room temperature under polyethylene sheeting. GC-1 and GC-2 were printed on 14 and 21 October 2025 and tested on 1 December 2025, at ages of 48 and 41 days, respectively. Before loading, both beams were rotated by 90° into their structural orientation. On the test day, GC-2 was loaded first, followed by GC-1. The printing process for GC-1 and GC-2 is shown in Figure 3 and Figure 4, respectively, and the actual reinforcement layouts are shown in Figure 5.

## 3. Results

### 3.1. Printing and Shape Quality of the Elements

The production of the GC beams verified the printing of a real, nearly three-metre-long reinforced element. At the same time, the insufficient shape stability of the mixture was confirmed.

#### Geometric Deviations and Data Limits

The achieved cross-sections, average bead widths, and approximate chord-diagonal joint widths are summarized in Table 4; both beams deviated substantially from the target geometry. GC-2 achieved a 170 mm section width compared with 140 mm for GC-1 (approximately 21% greater). The post-test joint widths were approximately 80 mm for GC-1 and 70 mm for GC-2, compared with 60 mm for the reference CC element. In every case, the joint width was approximately twice the corresponding average bead width (40, 35, and 30 mm, respectively). For the same 250 mm structural depth, the wider printed shell/chord geometry provides a more favorable section modulus and is therefore structurally relevant to the comparison of the four-point-bending results.

Layer-by-layer settlement, interlayer gaps, and longitudinal section profiles were not measured during printing. Because the GC joint-width values were read from irregular post-test surfaces, they provide dimensional context rather than exact pre-test geometry. At this proof-of-concept stage, completing the nearly 3 m print was itself the main challenge and required continuous operator adjustment.

Systematic geometric monitoring should follow once setting acceleration and the printing process are sufficiently repeatable.

### 3.2. Load-Displacement Diagrams and Load-Bearing Capacity

**Figure 6:**
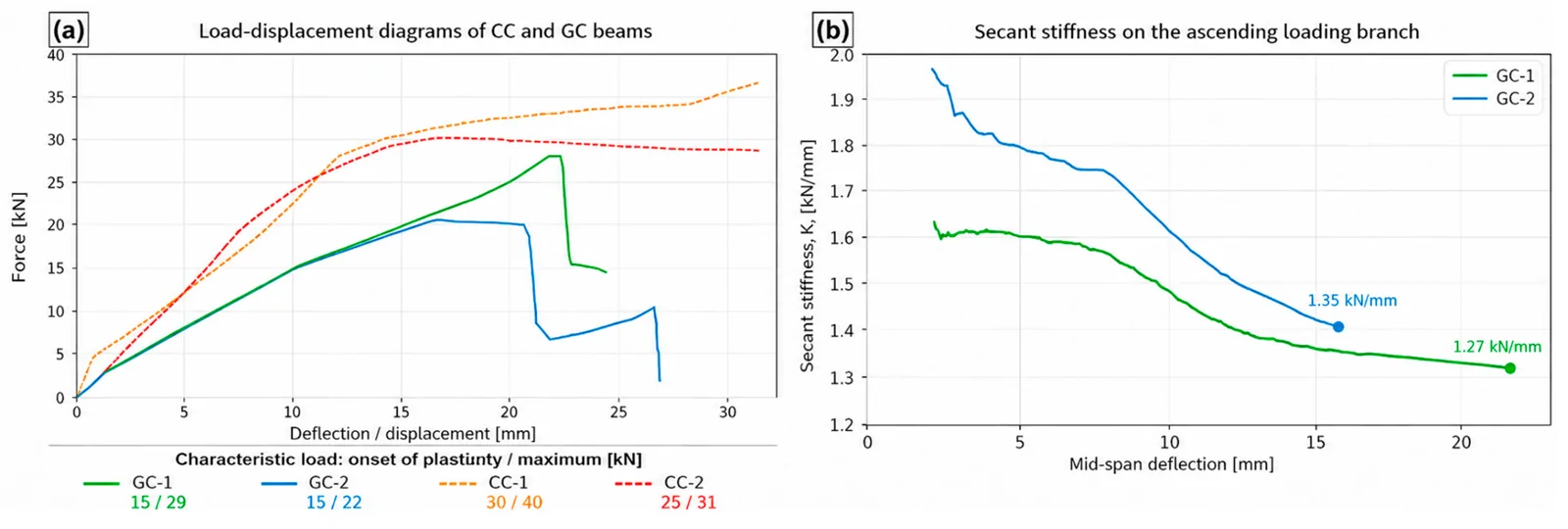
Mechanical response of the tested beams: (**a**) load-displacement diagrams of the CC and GC beams; (**b**) evolution of secant stiffness during the ascending loading branch of the GC beams.

Secant stiffness Ks = (F − F0)/(δ − δ0) was evaluated on the ascending GC branches from δ0 = 0.10 mm to peak load. Archived CC time series were unavailable, so Figure 6b is limited to GC-1 and GC-2.

**Figure 7:**
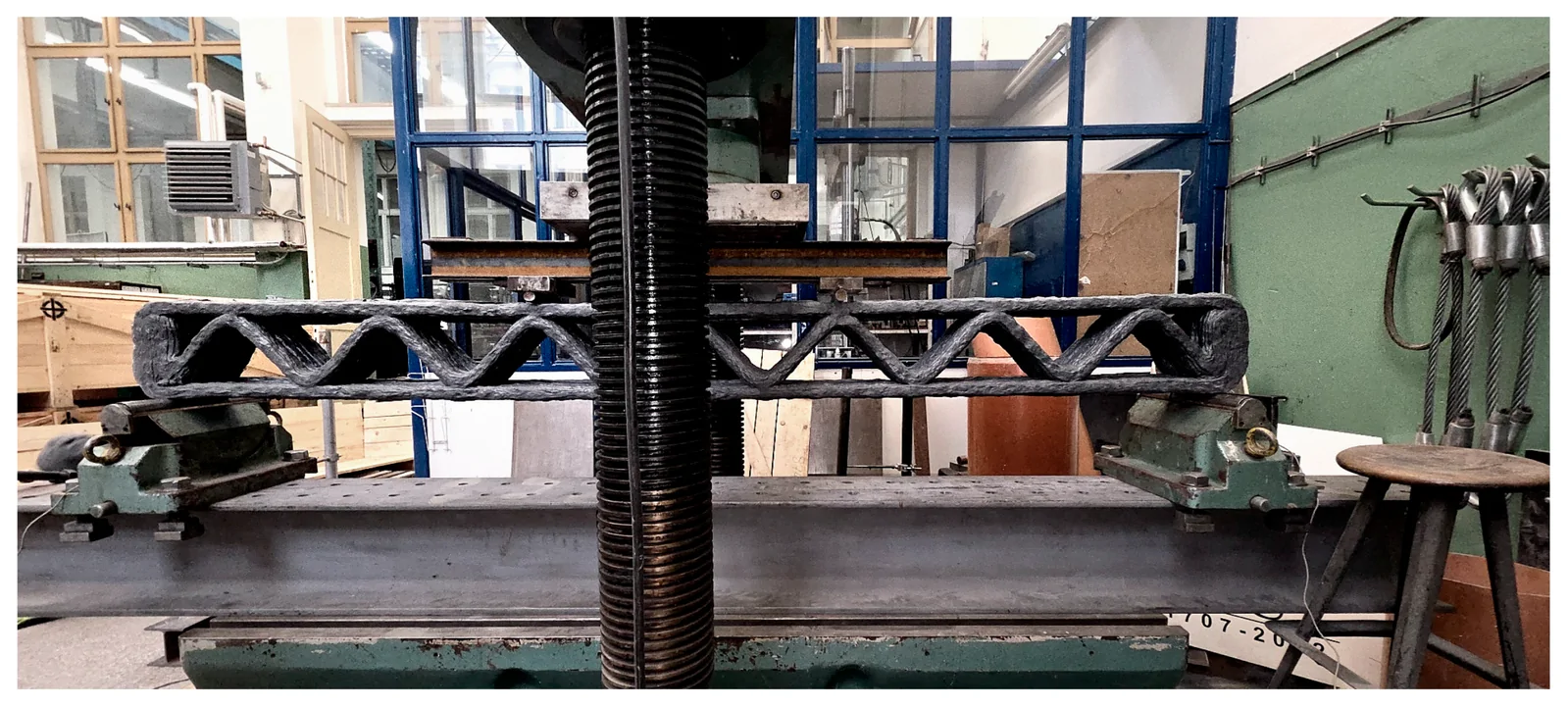
Four-point bending test of the GC beam.

Two GC beams were tested in four-point bending and reached peak loads of approximately 22 and 29 kN. GC-2 was loaded first and reached 21.61 kN; GC-1 was loaded second and reached 29.27 kN. Secant stiffness decreased from approximately 1.96 to 1.35 kN/mm in GC-2 and from 1.63 to 1.27 kN/mm in GC-1 over the evaluated ascending branch. Although GC-2 had the wider achieved section and received local hot-air treatment, GC-1 reached the higher peak force. The difference therefore cannot be attributed to section width or heating alone, and the two GC tests were not otherwise identical. CC retained reserve capacity up to reinforcement rupture; GC failed through node failure and loss of stability. The four-point bending test setup is shown in Figure 7, and the loading-test results are summarized in Table 5.

### 3.3. Failure Mode

The CC beams failed by rupture of the tensile reinforcement in the bottom chord; the layers and joints remained functional. The structure therefore utilised the reinforcement up to the ultimate limit state.

**Figure 8:**
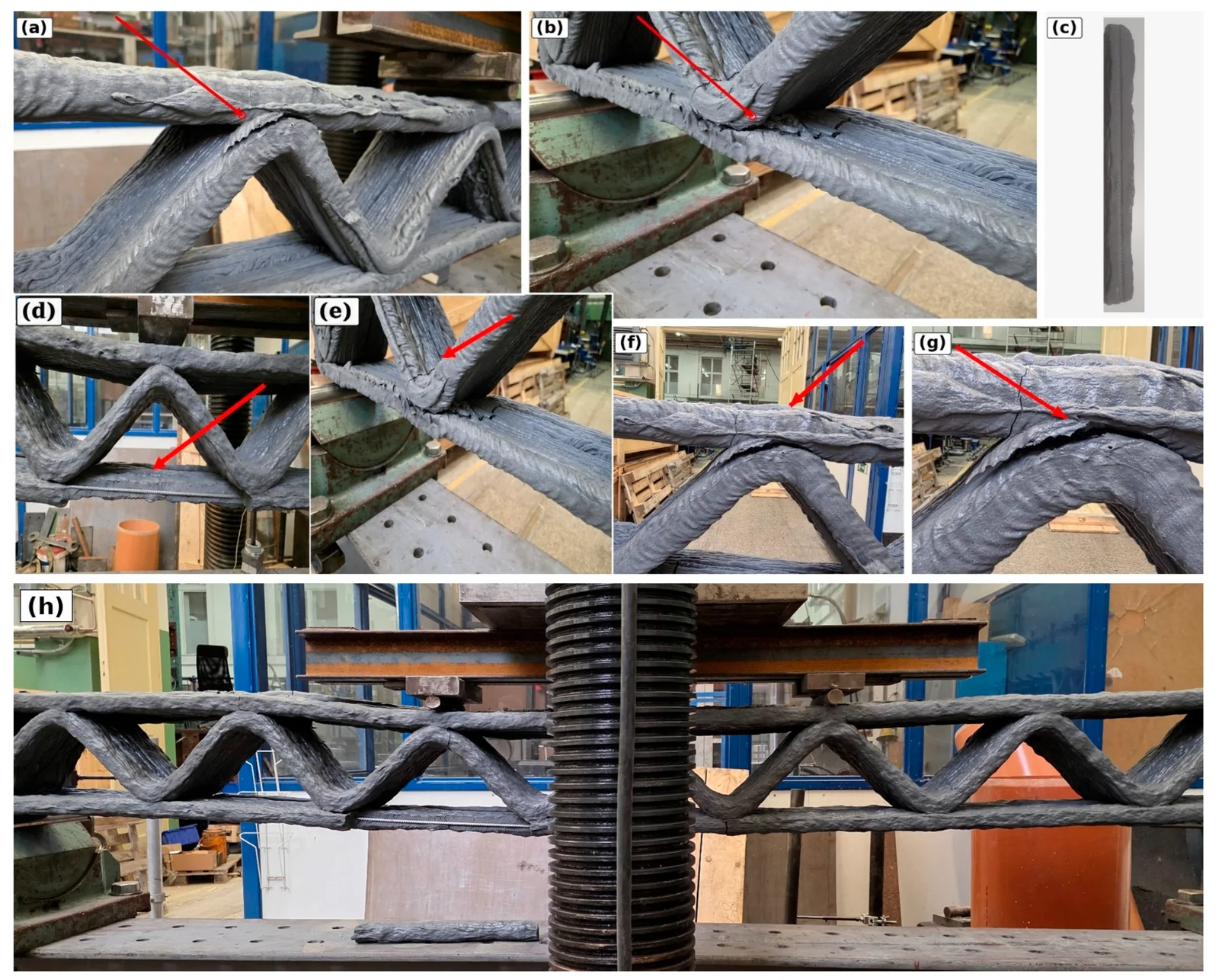
Failure documentation: (**a**) top-chord failure zone at the chord-diagonal interface; (**b**) bottom-chord failure zone at the chord-diagonal node; (**c**) bottom-chord fragment showing intact longitudinal reinforcement after matrix failure; (**d**) lower-chord fracture under load, with the arrow indicating the broken lower chord; (**e**) local lower chord-diagonal joint, with the arrow indicating the point where the diagonal meets the chord; (**f**) upper-node separation after failure; (**g**) detail of the upper-node crack/interface; (**h**) overall view of the failed beam during loading.

In the GC beams, the reinforcement did not rupture. The decisive process was joint failure, deformation, and local disintegration of the matrix, probably due to the combined effect of lower strength, low modulus of elasticity, higher shrinkage, weaker bond to reinforcement, and printing deviations. Representative failure zones, including an overall view of the failed beam, are documented in Figure 8.

#### Reinforcement-Matrix Interaction

Reinforcement strain and bond-slip were not measured (no strain gauges, DIC, or pull-out tests), so reinforcement-matrix interaction can be assessed only qualitatively. The B500B bars remained intact while the GC nodes and matrix failed. The approximate joint widths were about 80 mm for GC-1 and 70 mm for GC-2, compared with 60 mm for the reference CC element. In each case, the joint width was approximately twice the average bead width. Because the GC boundaries are irregular and were exposed only after failure, these values are approximate and are not statistical contact-width measurements.

**Figure 9:**
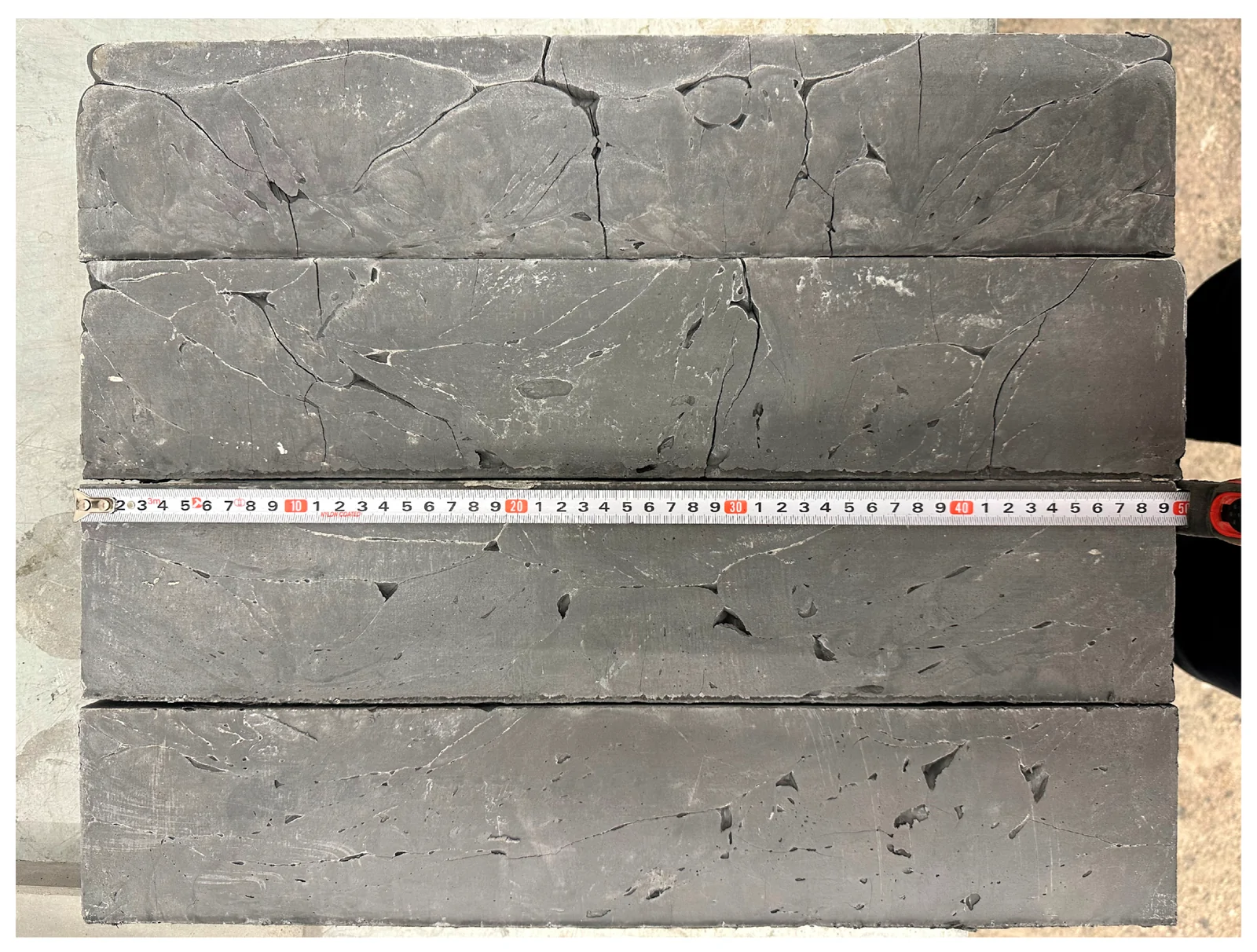
Four 500 mm companion specimens after shrinkage monitoring: two reinforced specimens on the left (four Ø6 mm bars) with many fine cracks and lower shrinkage, and two unreinforced specimens on the right with a shrinkage crack up to about 1 mm.

Four 500 mm companion specimens provided indirect shrinkage evidence. Two specimens reinforced with four Ø6 mm bars showed many fine cracks and lower shrinkage (reported 1.6 mm/m) than the two unreinforced specimens (7.1 mm/m), which developed a shrinkage crack up to about 1 mm (Figure 9).

**Figure 10:**
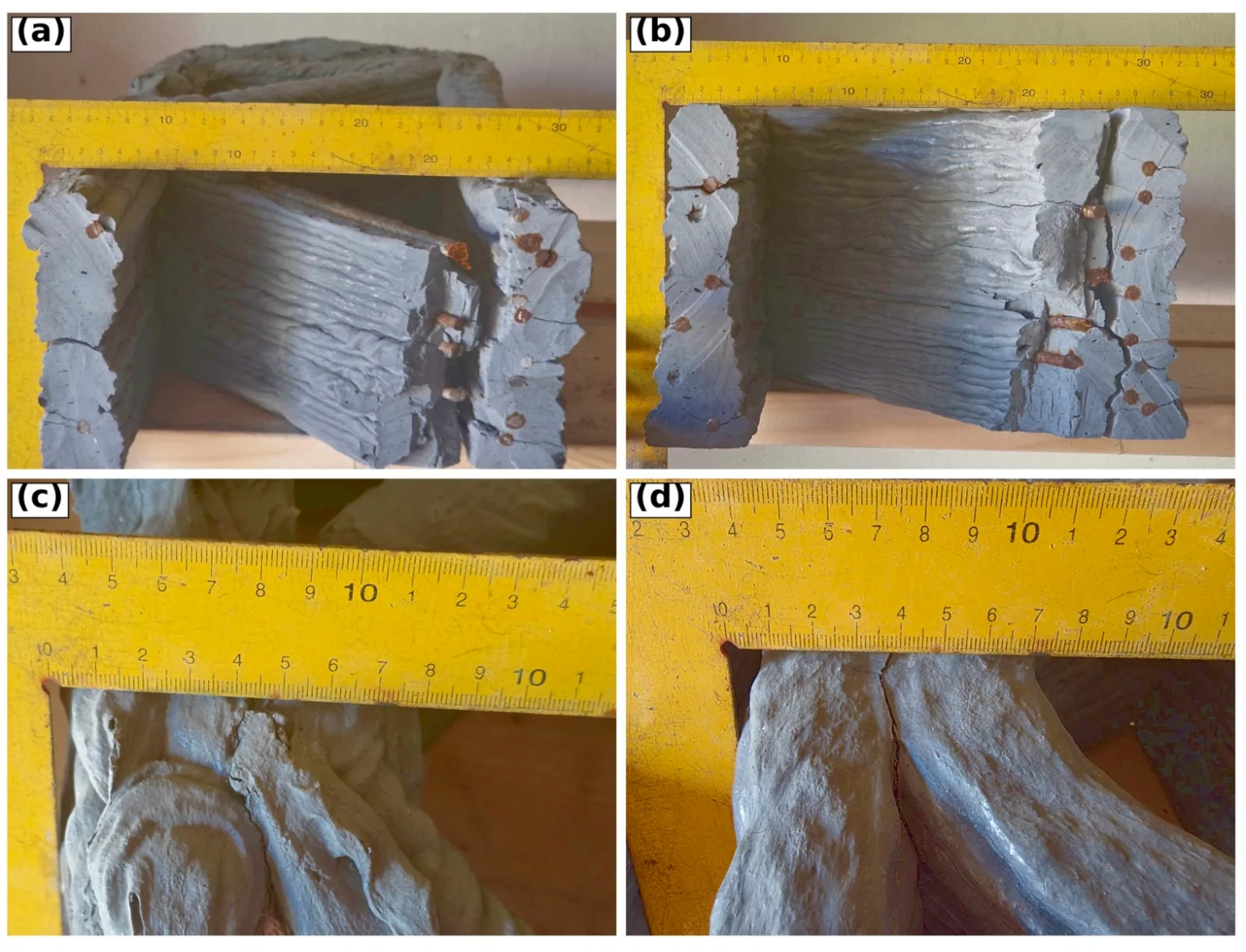
Post-test cross-sectional and joint documentation with enhanced tonal contrast for visibility: (**a**) overall cross-sectional view of GC-1 (achieved width approximately 140 mm); (**b**) overall cross-sectional view of GC-2 (achieved width approximately 170 mm); (**c**) exposed chord-diagonal joint in GC-1 with a metric ruler (approximate joint width 80 mm); (**d**) exposed chord-diagonal joint in GC-2 with a metric ruler (approximate joint width 70 mm). Panels (**a**,**c**) show GC-1 in the left column; panels (**b**,**d**) show GC-2 in the right column. The ruler-based observations are post-test and approximate because of the irregular fracture boundaries.

**Figure 11:**
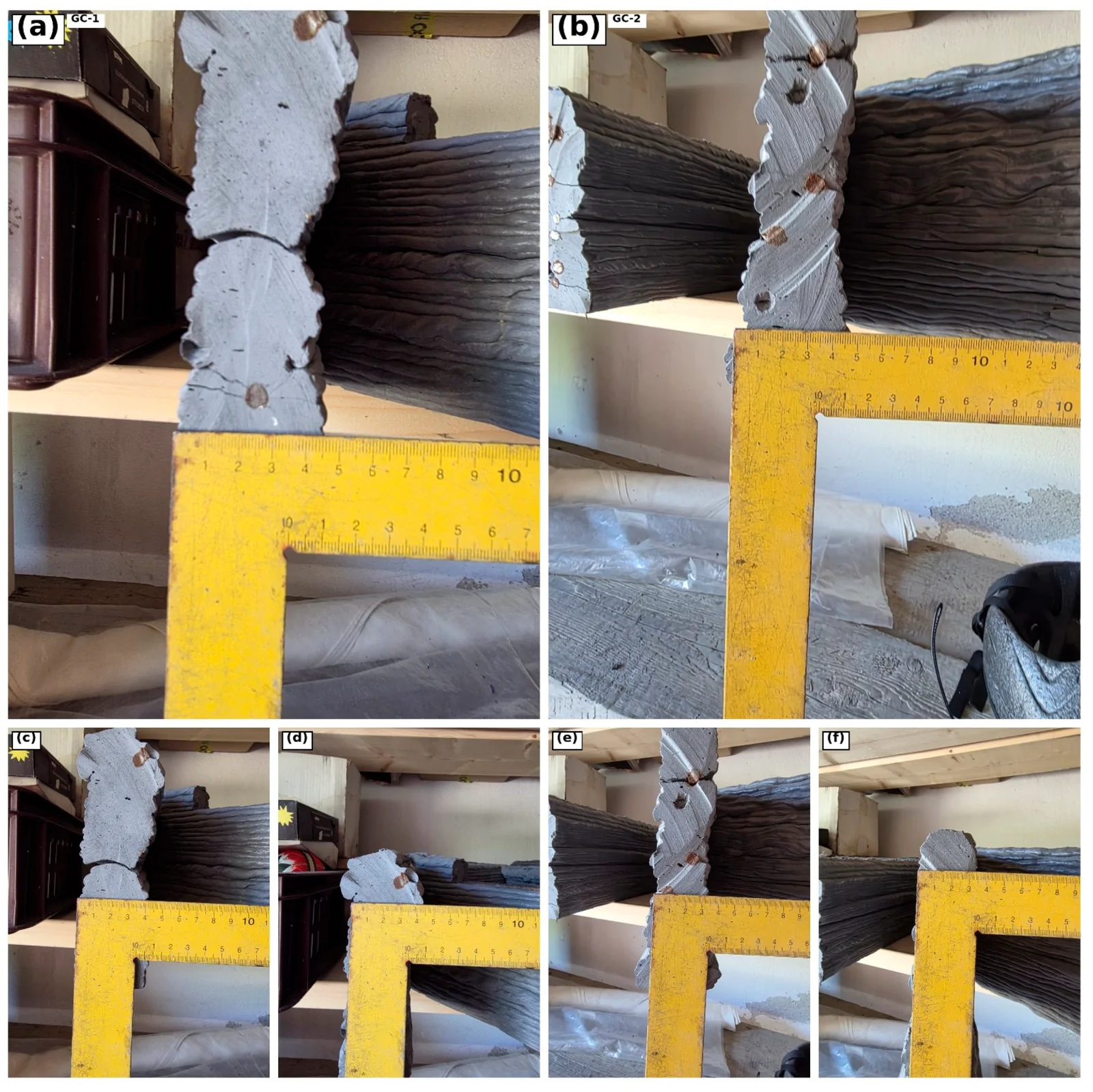
Post-test ruler-based documentation of exposed chord cross-sections: (**a**,**c**,**d**) GC-1; (**b**,**e**,**f**) GC-2. The original photographs are shown without changing their aspect ratios. The left edge of panel (**a**) is aligned with panel (**c**), the right edge of panel (**b**) is aligned with panel (**f**), and the inter-panel spacing is 2 mm. These images show sections through the chords, not chord-diagonal joints; therefore, no node-contact dimensions are inferred from them.

Future work should add replicate material tests, cross-layer tests, pull-out tests, and systematic geometric monitoring to separate matrix, interlayer, and reinforcement-bond effects. Post-test cross-sectional and joint documentation is provided in Figure 10 and Figure 11.

## 4. Discussion

### 4.1. What the Results Demonstrate

The experiment verified the printing, reinforcement, and loading of a GC beam at a scale of almost 3 m. Its contribution is the comparison of the structural response with the CC reference.

The data show differences in stiffness, load-bearing capacity, load-displacement response, and failure mode. With *n* = 2 per material system and different achieved GC sections, the ranges are descriptive only; no inferential statistics or general design rules are claimed. Within the GC pair, GC-2 and GC-1 had achieved sections of 170/250 mm and 140/250 mm, respectively. Despite the wider cross-sectional geometry of GC-2, it reached the lower peak force (22 kN versus 29 kN for GC-1). This countertrend shows that section geometry alone did not govern the observed difference. Because only one specimen was tested for each process condition and the printing parameters differed, no isolated effect of hot-air treatment or section width can be claimed.

Previous studies used continuous steel cable or microcable reinforcement in printed geopolymers [8,9]. Such reinforcement may improve crack control, but it cannot compensate for unstable bead geometry or weak nodes; these effects require separate verification.

### 4.2. Why the Difference in Load-Bearing Capacity and Stiffness Cannot Be Reduced Only to Compressive Strength

The lower load-bearing capacity and earlier nonlinearity of GC cannot be explained by a single strength value. At 28 days, GC reached approximately 41% of the CC compressive strength, 85% of the flexural tensile strength, 12% of the static modulus, and more than fourteen times the shrinkage. The decisive factor was the combination of low stiffness, high shrinkage, geometric inaccuracy, and weakened joints.

This conclusion is consistent with the literature on 3D printing of GC: mechanical performance is determined not only by strength, but also by the rheological window, structuration after extrusion, interlayer bonding, anisotropy, and sensitivity to process conditions [1,2,3,4,5,6,7]. Zhang et al. show that open time and the rheological window affect print quality, strength, and anisotropy [15]. We therefore interpret the failure mode as the combined effect of material and process factors.

### 4.3. Evaluation of the Role of Reinforcement

As shown in Section 3.2, para. 2 and Section 3.3, paras. 1–2, the reinforcement was less fully utilized in GC: the bars remained intact, whereas CC failed by reinforcement rupture.

The result does not imply that steel reinforcement is unsuitable. For a GC matrix with the given stiffness, shrinkage, and geometric precision, however, the reinforcement concept cannot be adopted from CC without modification. In digitally manufactured CC and GC elements, reinforcement must be understood as part of the manufacturing system, not merely as an inserted element from conventional reinforced concrete [8,9,10,11].

### 4.4. Technological Limit: Setting Acceleration After Layer Deposition

Manual heating is described in Section 2.4, para. 3; Table 4 summarizes the process parameters. The practical limit was not extrusion but strengthening after deposition. GC-2 reached the lower peak force (22 kN), despite its wider achieved section (170/250 mm versus 140/250 mm for GC-1) and hot-air treatment. This single comparison therefore provides no evidence of a structural benefit from manual heating. The difference cannot be attributed to heating alone because the nozzle diameter, printing speed, achieved geometry, and overall print regime also changed, and only one beam was tested per condition. The loading sequence was GC-2 first, followed by GC-1.

Manual hot-air heating produced an immediate small increase in surface cohesion and less local sagging; after about 20 min, lower heated layers felt stiffer. Because heating was applied manually and non-uniformly, it cannot be quantified as an independent process effect.

Whether hot-air heating is the best route remains open. Follow-up research should compare controlled hot air, infrared and microwave heating [16] with a non-thermal route using a GC-compatible chemical accelerator at the print head. The present treatment did not provide the repeatability required for a mature manufacturing process.

### 4.5. Material-Technological Implications of the Results

The supporting evidence is summarized in Table 3 and Table 4 and Section 3.3, para. 2. Matrix and node performance should be improved before reinforcement quantity is optimized.

For repeatability, future tests should record mixture temperature, ambient conditions, mixing time, open-head refill level, travel speed, spindle speed/power, bead geometry, layer settlement/gaps, and any accelerator or heating input. For practical use, however, the mix must also tolerate ordinary variation in temperature, humidity, and timing rather than depend on narrowly controlled laboratory conditions.

The central question of this study was deliberately narrow: can a load-bearing GC structural element be 3D printed and mechanically tested? The answer is yes. The nearly 3 m reinforced truss was printed and tested in four-point bending. This proof-of-concept (*n* = 2) opens further questions on repeatability, rheology, setting acceleration, node geometry, reinforcement-matrix interaction, and scale-up; answering them requires a much larger experimental programme and budget.

## 5. Conclusions

- A GC beam 2.93 m long can be 3D printed, reinforced, and loaded in four-point bending.
- The GC beams reached maximum forces of 22 and 29 kN; the CC references reached 31 and 40 kN. GC-2 (22 kN) was loaded before GC-1 (29 kN). Although GC-2 had the wider achieved section (170/250 mm versus 140/250 mm) and received hot-air treatment, it reached the lower peak force. With one beam per process condition and different nozzle, speed, and geometry parameters, the difference cannot be assigned to hot-air heating or section width alone.
- Nonlinearity occurred in GC at approximately 15 kN and in CC at 25–30 kN; CC had higher stiffness and reserve capacity after reinforcement yielding.
- CC failed by rupture of the tensile reinforcement; GC failed by joint failure, deformation, and local disintegration of the matrix.
- The difference cannot be explained by compressive strength alone; low modulus, high shrinkage, geometric deviation, and node weakness also governed the response (see Section 4.2, para. 1).
- The B500B reinforcement was not fully activated at GC failure; matrix, node, and bond performance should be improved before reducing the reinforcement ratio.
- The main limit was fresh-state shape stability and the absence of robust, reproducible post-deposition stiffening.
- For the next prototype stage, process targets are recovery of the intended 200/250 mm section and an average bead width close to 30 mm; these are development targets, not design rules.
- The study answers its core question affirmatively: a nearly 3 m reinforced GC structural element can be printed and mechanically tested. The remaining process questions require a larger follow-up programme.

## Figures and Tables

**Table 1:** Composition and preparation of the GC mixture used.

Component	Ratio [-]
Baucid	1.00
Water glass	0.90
Carbon fibres	0.01
Cellulose	0.01
3M K15 Glass Bubbles	0.04
Graphite	0.05
Agar	0.005
Xanthan gum	0.005
Anhydrite	0.005
Mixing method and equipment	industrial dough mixer; minimum experimental batch of 50 kg of wet mixture
Specimens and conditions	flexure/modulus/shrinkage: approx. 40 × 40 × 160 mm; compression: approx. 100 mm cubes; shrinkage: 20 ± 1 °C, RH 50 ± 5%

**Table 4:** Printing parameters of the GC beams and the reference CC element.

Parameter	GC-1	GC-2	Ref. CC	Note
Nozzle diameter [mm]	15	25	–	
Approx. speed [m/min]	1.3–2.9	0.9–2.1	–	adjusted to mixture fluidity
Nominal height [mm]	200	200	200	dimension during printing; after 90° rotation, resulting section width
Actual height [mm]	140	170	200	effect of mixture spreading
Required bead [mm]	30	30	30	same as reference CC element
Average bead [mm]	40	35	30	
Approx. chord-diagonal joint width [mm]	≈80	≈70	60	≈2 × the corresponding average bead width in every case
Printing time [min]	120	150	–	
Acceleration during printing	no	hot air (Section 2.4, para. 3)	chemical (Section 2.3, para. 1)	

**Table 5:** Summary of the results of the loading tests of the truss beams.

Beam	Section [mm]	Bead [mm]	Failure	Force at Plastic Onset [kN]	Max. Force [kN]	Deflection at Plastic Onset [mm]
CC–1	200/250	30	Rebar rupture	30	40	13
CC–2	200/250	30	Rebar rupture	25	31	12
GC–1	140/250	40	GC failure	15	29	8
GC–2	170/250	35	GC failure	15	22	8
CC mean (range)	200/250	30	Rebar rupture	27.5 [25–30]	35.5 [31–40]	12.5 [12–13]
GC mean (range)	155/250	37.5 [35–40]	GC joint failure	15.0 [15–15]	25.5 [22–29]	8.0 [8–8]
GC/CC [%]	–	–	–	54.5	71.8	64.0

Note: Bracketed values are descriptive min-max ranges for each material system (*n* = 2); they are not inferential statistics.

## Data Availability

The original contributions presented in this study are included in the article. Further inquiries can be directed to the corresponding authors.
